# Commentary: Anti-hyperplasia effects of total saponins from phytolaccae radix in rats with mammary gland hyperplasia via inhibition of proliferation and induction of apoptosis

**DOI:** 10.3389/fphar.2023.1181730

**Published:** 2023-05-22

**Authors:** Liying Cai, Guoxin Sun, Jie Cheng

**Affiliations:** ^1^ College of Nursing and Rehabilitation, North China University of Science and Technology, Tangshan, Hebei, China; ^2^ School of Clinical Medicine, Qingdao University, Qingdao, Shandong, China; ^3^ Affiliated Hospital of North China University of Science and Technology, Tangshan, Hebei, China

**Keywords:** mammary gland hyperplasia, toxicity tests, total saponins of phytolaccae, dose optimization, pharmacodynamic

## Introduction

Mammary gland hyperplasia (MGH) is a non-tumor, non-inflammatory proliferative breast disease that increases the risk of breast cancer. Early treatment and management can prevent breast cancer, but current treatment methods such as hormone medications and surgery have limitations, including adverse reactions and changes in breast appearance. Therefore, safe and effective treatment methods are needed to address these limitations.

*Phytolacca acinosa* Roxb and *Phytolacca americana* L are perennial herbaceous plants belonging to the Phytolaccaceae family, commonly found in tropical or temperate regions like Assam, North-Central China, Japan, Korea, East Himalaya, Vietnam, Arizona, California, and Mexico (Plants of the World Online, 2023a; Plants of the World Online, 2023b). Phytolaccae Radix, a medicinal material, contains diverse chemical compounds such as saponins, flavonoids, phenolic acids, sterols, and polysaccharides, which have been shown to exhibit anti-inflammatory (Liu et al., 2022) and anti-tumor (Jung et al., 2015) activities. Moreover, Phytolaccae Radix has been found to be effective in the treatment of MGH and endometriosis (Gao et al., 2009).

Li et al. (2018), conducted a study in which they treated estrogen and progestogen induced MGH in rats with total saponins of Phytolaccae (TSP). After 1 month of treatment, the researchers observed a significant reduction in nipple swelling in the treated rats. Pathological examination revealed that TSP were as effective as tamoxifen in inhibiting the proliferation of mammary epithelial cells, and they were significantly more effective than the MGH model group. In addition, TSP were found to regulate serum sex hormones levels in MGH rats, inhibit the expression of ERα, PR, bFGFh, and VEGF proteins, induce proliferation cell apoptosis, and exhibit anti-breast hyperplasia effects.

## Room for improvement

Although the study yields promising results, there are some limitations that require attention. Firstly, the description of the extraction process of TSP was insufficiently detailed. The source and quantity of plant roots should be disclosed to ensure the reproducibility of experimental results. Additionally, the degree of grinding of plant particles should be clearly stated as it directly affects the concentration of TSP extracted.

Secondly, the authors conducted an analysis of the extract’s composition and discovered the presence of 19 substances. Despite triterpene glycoside being the primary component, there were still other substances detected. Therefore, there is a need for further improvement in serum drug testing to identify the specific substance responsible for the observed effects, which would facilitate further research.

Thirdly, it is necessary to conduct toxicology experiments (Nguyen et al., 2021). Although the efficacy of TSP increased gradually with the dose in the experimental group, a high concentration of total saponins (30 mg/kg) did not show significant superiority over the positive control group (1.5 mg/kg tamoxifen). Conducting further toxicology experiments to establish the safe dosage of medication and setting concentration gradients within the safe dosage range to test the optimal therapeutic concentration would be beneficial. Further increasing the dosage within the safe dosage range may result in better outcomes.

Despite the limitations of this study, the conclusions have important reference and guidance significance. As the authors have stated, TSP are an effective medication that can significantly alleviate symptoms of MGH. Furthermore, researchers should develop more alternative therapies for patients with MGH to enhance their quality of life.
